# Workaholism: An overview and current status of the research

**DOI:** 10.1556/JBA.2.2013.017

**Published:** 2013-12-06

**Authors:** Cecilie Schou Andreassen

**Affiliations:** Department of Psychosocial Science, University of Bergen, Bergen, Norway The Bergen Clinics Foundation, Bergen, Norway

**Keywords:** antecedents, intervention, measurement, prevalence, outcomes, workaholism

## Abstract

*Aims:* This article addresses the stable tendency of excessive and compulsive working (i.e., workaholism). The main aim is to provide an updated oversight of the research area related to definition, prevalence, assessment, causes, outcomes, intervention as well as proposed future research directions. The target-population is both researchers and clinicians. *Methods:* The findings are identified by narratively reviewing the literature. *Results:* Research into workaholism has expanded over the last two decades. Several screening instruments to identify workaholics have been developed. The vast majority of these are based on seemingly atheoretical foundations, lacking convergent validity with each other and with related constructs. Research generally shows that workaholism is related to impaired health and well-being as well as to conflicts between work and family life. Workaholism is probably caused and maintained by a range of factors, although solid empirical underpinnings for suggested antecedents are currently sparse. So far no well-evaluated interventions for workaholism exist. *Conclusions:* At present, workaholism as a construct lacks conceptual and empirical clarity. Future research efforts should prioritize longitudinal studies as well as studies incorporating unbiased, firm parameters of both health and behavior.

## Introduction

Work is ordinary and necessary for most people and provides us several positive things. It gives us salary, sets the day, gives us a sense of who we are, forms relationships, and gives us purpose. In spite of the many positive aspects of work, however, some people are seemingly driven by internal and external forces to work excessively and compulsively. These people are often called workaholics (Schaufeli, Taris & Van Rhenen, 2008). Workaholism may have contradictory psychological, physical, and social effects/outcomes for the person in question and for those closest to him/her. It may also negatively affect the work environment.

**Figure 1.**
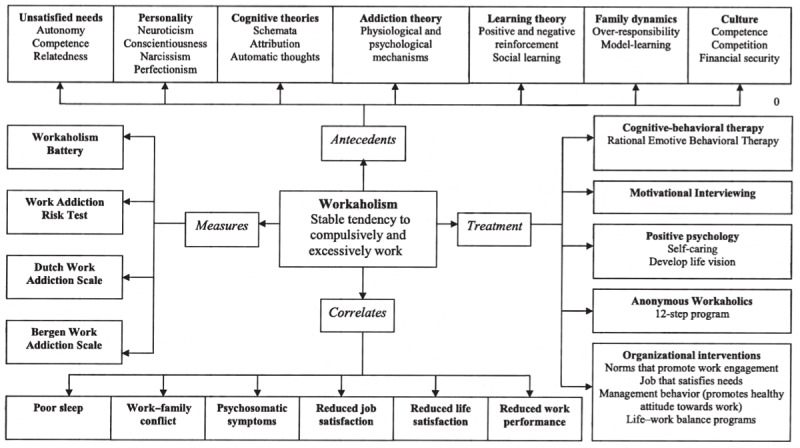
Schematic overview of the workaholism field, including particular measurements, possible antecedents and consequences (correlates) of workaholism, and potential treatment approaches

This article provides an overview of workaholism and the current status of research in this field. Theoretical as well as practical aspects of this timely and topical phenomenon are highlighted (see Figure 1). I begin by discussing workaholism as a construct. After approaching measurements, I focus on key theories and empirical analyses that encompass conceivable causes and consequences of workaholism. Thereafter, implications of present knowledge for intervention on both the individual and organizational level are described. Finally, I point out areas where more knowledge is needed.

## The Workaholism Construct

*Workaholism* as a construct stems originally, by analogy, from the term *alcoholism.* It was first defined as “addiction to work, the compulsive and uncontrollable need to work incessantly” (Oates, 1971). In roughly 40 years, the term has become a part of colloquial language. Research into this topic has also increased during the past two decades, and the terms *work addiction, workaholism,* and *excessive overwork* have been used interchangeably (Andreassen, 2013). Some of the first definitions narrowed the phenomenon to those who work more than 50 hours a week (Mosier, 1983). It is apparent that a large portion of today’s workforce could easily fit into this definition, as the average (non-workaholic) management-level worker devotes at least 50 hours per week to work (Brett & Stroh, 2003). Other researchers more broadly described workaholics as those who always invest more time and energy in work than what is required (Machlowitz, 1980). This definition takes into account the workaholic’s attitude towards work, rather than merely the time spent on work, and is more in line with the modern understanding of the construct. Most contemporary definitions describe workaholism as a continual pattern of high work investment, long working hours, work beyond expectations and an all-consuming obsession with work (Griffiths, 2011; Ng, Sorensen & Feldman, 2007). Some perspectives remain controversial. Certain researchers hold that workaholism is a positive attribute by emphasizing the benefits gained from heavy work investment (e.g., extra work efforts), while most scholars have highlighted the negative and riskier sides (e.g., impaired health and work–life conflicts) (Griffiths, 2011; Oates, 1971; Robinson, 2013; Schaufeli, Shimazu & Taris, 2009).

Workaholic typologies have rarely been based on solid theoretical or empirical underpinnings. Scott, Moore and Miceli (1997) proposed three types of workaholics: *the compulsive-dependent, the perfectionists* and *the achievement-oriented.* Robinson (2013) portrayed four workaholic types: *the bulimic* who make it a point to do the job perfectly or not at all; *the relentless* who are compulsively driven to work quickly and meet deadlines, and who find it difficult to stop working; *the savoring* who are consumed by a preoccupation with details; and *the attention-deficit* who start numerous projects/ventures but become easily dulled and restless, continually motivated to seek further challenges. One empirically underpinned distinction is between workaholics who appreciate their job and those who do not (Bonebright, Clay & Ankenmann, 2000). Specifically, Spence and Robbins (1992) discovered *enthusiastic workaholics* who are signified by elevated levels of work involvement, driven by an inner compulsion to work, and who find great joy and contentment in work; and *non-enthusiastic workaholics* who are similarly work-involved and internally driven, but who appear to derive little pleasure from excessive work. The non-enthusiastic types were originally termed *real* workaholics, whereas the designation *non-enthusiastic* was eventually added to the typology (Bonebright et al., 2000). Despite this seemingly simple distinction between types of workaholism, the classification is useful, topical and meaningful.

In their 2007 review of this research field, Ng et al. (2007) underlined the significance of discriminating between gratification from the job itself and gratification from the *act of working.* The authors argued that all workaholics are gratified by the act of working, as it tranquilizes and neutralizes uncomfortable moods, emotions and sensations otherwise felt when not working (feelings of anxiety, guilt) (i.e., Drive, “I feel guilty when I take time off work”). Note that Work Enjoyment is used as one of the conditions in the widely used Workaholism Battery (WorkBAT; Spence & Robbins, 1992) where elevated scores identify enthusiastic workaholics primarily as those who truly appreciate the work they do (e.g., “I do more work than is expected of me strictly for the fun of it”). However, the dual terms enthusiastic and non-enthusiastic workaholism are currently probably better covered by the terms work engagement and workaholism (Taris, Schaufeli & Shimazu, 2010). Classification of these particular work attitudes and behaviors is still the subject of ongoing debate, as some scholars have recently re-encouraged usage of the distinction between *workaholics, work engaged workaholics* and *engaged workers* (van Beek, Taris & Schaufeli, 2011).

Spence and Robbins (1992) understand workaholism as a threefold construct where *real* workaholics typically score high on work involvement, high on work drive, and low on work enjoyment. This multidimensional model has been criticized. Firstly, some scholars have argued that work enjoyment is irrelevant for the purpose of defining workaholism (Mudrack, 2006). Secondly, empirical studies consistently conclude that the work involvement subscale is invalid (Andreassen, Hetland & Pallesen, in press). Furthermore, recent research operationalizes workaholism predominantly from dimensions that correlate highly (Schaufeli et al., 2009) or that perceive the construct as unidimensional (Andreassen, Griffiths, Hetland & Pallesen, 2012). Hence, the understanding of workaholism as a multidimensional construct has apparently had to give way to a more unidimensional understanding that first and foremost gives priority to the drive dimension as the *heart* of workaholism. Theoretically speaking, there seems to be striking similarities between the construct of workaholism and constructs like obsessive passion towards work (Vallerand, Paquet, Philippe & Charest, 2010) and work overinvolvement (Lehr, Koch & Hillert, 2010). Whether these constructs merely redefine workaholism in new, modern terms (new wine in old bottles) or truly bring something new to the table is currently unclear.

Since the term workaholism was introduced in the academic literature, scholars have variably perceived it as an attitude, a trait, an obsession and/or compulsion, and as an addiction. Recently, the initial addiction approach has been given credence (Andreassen, 2013; Sussman, 2012). Thus, from the perspective of an addiction, workaholism may be described as “being overly concerned about work, to be driven by an uncontrollable work motivation, and to put so much energy and effort into work that it impairs private relationships, spare-time activities and/or health” (Andreassen et al., in press). Workaholism may be experienced subjectively as a loss of control, where the workaholic continues to engage in work despite acknowledged negative consequences.

Importantly, workaholism differs from behaviors such as bipolar conditions. *DSM-5* criteria for a manic episode include, among other factors, excessive engagement in activities with vast potential for detrimental outcomes (American Psychiatric Association [APA], 2013). Excessive engagement in work activity is merely one incident of manic behavior, and far from all bipolars are workaholics. This also applies for attention deficient hyperactivity disorder (ADHD). Moreover, workaholism is seen as a rather stable personal behavioral tendency – a tendency that most certainly can be exacerbated by the opportunities found in new technological innovations (laptops, smartphones, the Internet). Thus workaholism differs from extra work effort due to inner (e.g., acute need for money) or outer (e.g., huge order demand) situational factors (Snir & Harpaz, 2012). Quite recently workaholics were distinguished from three other groups with apparently the same chronic high notch work investment tendency (Snir & Harpaz, 2012). These were labeled the *work-devoted,* the *intimacy-avoiders* who work excessively to avoid intimacy/affection, and the *leisure-uninterested* who work much to fill their otherwise empty spare-time. This differentiation remains unexplored.

Taken together, from a differential diagnostic perspective, it is crucial to preclude possible medical conditions that may cause excessive work behavior. Also, one should recognize that not all excessive working is pathological.

## Prevalence

In a recent article it was estimated that approximately 10 percent of the general U.S. population may be workaholics (Sussman, Lisha & Griffiths, 2011). In other studies, the estimates have sometimes been significantly higher (Andreassen, Griffiths, Hetland et al., 2012). New research shows that workaholism is more prevalent among management-level workers and in specific sectors (agriculture, construction, communication, consultancy, commercial trade) (Andreassen, Griffiths, Hetland et al., 2012; Taris, van Beek & Schaufeli, 2012). It must be added, however, that we currently have little specific knowledge about the number and type of people affected by workaholism. Three reasons for this are (1) a lack of consensus on how workaholism should be defined, (2) a lack of a common understanding about how workaholism should be measured and where the cutscore between *normal* and *pathological* work behavior should be set, and (3) a nearly total lack of surveys in the field that have used representative samples.

## Measurement

In the 1980s researchers began to develop screening measures to identify workaholism. Some of these are more widely used and thoroughly evaluated than others. This section presents all measures mentioned in the literature to date (see Table 1 for particular measures).

The *Workaholism Battery* (WorkBAT) was developed by Spence and Robbins in 1992 and has been used in approximately 500 studies, making it the most widely used workaholism measure so far (Patel, Bowler, Bowler & Methe, 2012). The WorkBAT consists of 25 items distributed along three subscales – *Work Involvement* (eight items; e.g., “I spend my free time on projects and other activities”), *Drive* (seven items; e.g., “I seem to have an inner compulsion to work hard”), and *Work Enjoyment* (ten items; e.g., “Sometimes I enjoy work so much I have a hard time stopping”). All items are scored along a 5-point Likert format continuum ranging from *strongly disagree* (1) to *strongly agree* (5). These subscales are sometimes called the workaholism triad, reflecting the authors’ definition of the phenomenon. Construction and theoretical underpinnings of the WorkBAT were based on workaholism attributes in the literature and the authors’ own hypotheses (Spence & Robbins, 1992). Initially WorkBAT was tested among students – where the items referred to schoolwork. Thereafter psycho-metrical testing was based on data from 291 U.S. social workers (134 men; median age 43 for men, 40 for women) with academic positions participating in a national mail survey – randomly selected from a National Association of Social Workers database. Calculation of internal consistencies demonstrated Cronbach’s alphas (male/female) of .69/.67 for Work Involvement, .81/.67 for Drive, and .86/.86 for Work Enjoyment. A cluster analysis showed that two types of workaholics emerged. Those scoring above the mean on all three subscales were called enthusiastic workaholics; whereas those scoring above mean on Work Involvement and Drive, and below the mean on Work Enjoyment were denoted as non-enthusiastic workaholics. Workaholics scored higher than work enthusiast and others on perfectionism, non-delegation of responsibility, job stress, and health complaints in the scale-construction study (Spence & Robbins, 1992). Different WorkBAT-versions exist (Buelens & Poelmans, 2004; Huang, Hu & Wu, 2010; McMillan, Brady, O’Driscoll & Marsh, 2002). While the WorkBAT has been widely adopted by researchers, follow-up studies have shown only partially good psychometric properties – because the Work Involvement subscale has consistently proven inadequate (Andreassen, Ursin & Eriksen, 2007; Kanai, Wakabayashi & Fling, 1996; McMillan et al., 2002). Although showing acceptable psychometrics, the Work Enjoyment subscale has also been criticized for having little relevance for the definition of workaholism (Mudrack, 2006).

In 1989, Robinson developed the *Work Addiction Risk Test* (WART). The WART is also a popular measure, having been used in approximately 150 studies so far (Patel et al., 2012). The scale consists of 25 items answered on a 4-point Likert scale ranging from *never true* (1) to *always true* (4). Symptoms recognized by clinicians treating workaholics represent the item-pool – viewing workaholism as an addictive behavior. Reliability and concurrent validity were tested in a sample of 363 U.S. students (107 men; mean age 22.0). Internal consistency was high (*a* = .88), and WART scores correlated with generalized anxiety (*r* = .40) and Type-A behavior (*r* = .37–.50) (Robinson, 1999). Satisfactory 2-week test–retest reliability was found in a sample of 151 U.S. students (*r* = .83) (Robinson, Post & Khakee, 1992), whereas split-half reliability (*r* = .85) was tested in a sample of 442 U.S. students and members of Workaholic Anonymous (Robinson & Post, 1995). The WART is often scored as a one single factor, but factor analysis of the data has identified three to five factors – *Compulsive Tendencies* (nine items; e.g., “I seem to be in a hurry and racing against the clock”), *Control* (seven items; e.g., “Things just never seem to move fast enough or get done fast enough for me”), *Impaired Communication/Self-Absorption* (five items; e.g., “I dive into projects to get a head start before all the phases have been finalized”), *Inability to Delegate* (one item; “I prefer to do most things myself rather than ask for help”), and *Self-Worth* (two items; e.g., “It is important that I see concrete results of what I do”) (Flowers & Robinson, 2002). Overall scores of 67–100 characterize highly workaholic tendencies; scores of 57–66 characterize moderately workaholic tendencies. Scores below 57 are regarded as indicating non-workaholic status. WART has been criticized by some researchers because it has not been deemed applicable for contemporary definitions of workaholism, and it seems to reflect Type-A behavior and anxiety rather than workaholism (Mudrack, 2006; Robinson, 1999).

The *Dutch Work Addiction Scale* (DUWAS) was recently developed by Schaufeli et al. (2009). They argued that workaholics typically devote much time to work, and are also obsessed by it. In line with this, two subscales that measure these phenomena were constructed. The DUWAS consists of 10 items, but a 17-item version also exists. It uses five items from the WART-Compulsive Tendencies (Robinson, 1989) denoted as *Working Excessively* (WE) (e.g., “I find myself still working after my co-workers have called it quits”), four items from the WorkBAT-Drive (Spence & Robbins, 1992) and one from the WART-Compulsive Tendencies which are grouped under the category of *Working Compulsively* (WC) (e.g., “It is hard for me to relax when I am not working”). All items are scored along a 4-point Likert scale ranging from *(almost) never* (1) to *(almost) always* (4). DUWAS was tested/constructed in a convenience sample of Dutch (*n* = 7,594; 48% men; mean age 36.4 [*SD* = 9.5]) and Japanese (*n* = 3,311; 51% men; mean age 34.4 [*SD* = 10.5]) workers recruited via their organizations or the Internet (Schaufeli et al., 2009). The sample represented different occupational groups. Exploratory Factor Analysis (EFA) based on data from half of the samples yielded two factors. Confirmatory Factor Analysis (CFA) based on data from the other half of the samples confirmed the two-factor solution (Root Mean Square Error of Approximation [RMSEA] = 0.06, Comparative Fit Index [CFI] = .91). Cronbach’s alphas in the Dutch sample for WE and WC were .78 and .73, respectively; and .73/.68 in the Japanese sample. Significant correlations were found between overwork and the DUWAS subscales in both samples (Dutch sample: *r* = .40/.23 [WE/WC]; Japanese sample: *r* = .53/.25). The scales were also concluded distinct from burnout and engagement (Schaufeli et al., 2009). Norms from a national Dutch database suggest that scores above >75th percentile on both subscales should be considered as workaholism (Schaufeli, van Wijhe, Peeters & Taris, 2011). Studies have shown good psychometric properties and a replicable factor structure of the DUWAS (del Libano, Llorens, Salanova & Schaufeli, 2010; Schaufeli et al., 2009).

The latest addition to the toolbox is the *Bergen Work Addiction Scale* (BWAS) developed by Andreassen, Griffiths, Hetland et al. (2012). They argued that most of the already established workaholism measurements have not been cross-validated against each other and that very few, if any, seem to be based on a well-defined theoretical foundation. Thus, anchored in general addiction theory, the BWAS operationalizes workaholism as being comprised of seven core addiction components including (1) *Salience* – preoccupation with work (“Thought of how you could free more time to work?”), (2) *Mood modification* – work to escape or avoid dysphoria (“Worked in order to reduce feelings of guilt, anxiety, helplessness, and/or depression?”), (3) *Conflict* – work comes in conflict with one’s own and others’ needs (“Down-prioritized hobbies, leisure activities, and/or exercise because of your job?”), (4) *Withdrawal* – dysphoria when prohibited from working (“Become stressed if you have been prohibited from working?”), (5) *Tolerance* – work increasingly more to achieve the same mental and physiological effect (“Spent much more time working than initially intended?”), (6) *Relapse* – falls back into old pattern after a period of improvement (“Experienced that others have told you to cut down on work without listening to them?”), and (7) *Problems* – work so much that health is negatively affected (“Worked so much that it has negatively influenced your health?”). Hence, the BWAS consists of seven items worded in line with diagnostic addiction criteria (APA, 1994, 2013; World Health Organization, 1992). All questions are scored along a 5-point Likert scale ranging from *never* (1) to *always* (5) asking how often during the last year the symptoms have occurred. Scoring “often” or “always” on 4 out of 7 components indicates workaholism. BWAS was constructed in a sample of 12,137 Norwegian workers from a variety of professions. Sample 1 (*n* = 11,769; 7,596 men; mean age, 40.4 [*SD* = 11.6]) was recruited from a television broadcast; Sample 2 (*n* = 368; 175 men; mean age, 46.4 [*SD* = 10.1]) from a second wave of a longitudinal web-based survey about working life. Selection of items was based on data from half of Sample 1 (*n* = 5,932). The assumed one-factor solution of the final 7-item scale was confirmed by CFA based on data from the other half of Sample 1 (RMSEA = 0.08, CFI = .96, Tucker–Lewis Index = .95). Cronbach’s alphas were .84 (Sample 1) and .80 (Sample 2). The suggested cut-off for categorization of workaholics demonstrated discriminative ability with respect to working hours, leadership position, and subjective health complaints. The BWAS score was positively associated with scores on WART (*r* = .50–.84) and WorkBAT (*r* = .35–.65), but seemed less related to the WorkBAT-Enjoyment subscale (*r* = .13). In a recent examination of *study addiction* (*N* = 218), BWAS was modified using the word *studying* instead of *work* (Andreassen, Griffiths et al., 2013). Internal consistency was acceptable (*α* = .74). Study addiction was positively associated with neuroticism (*β* = .41) and conscientiousness (*β* = .24) as well as exercise (*r* = .27) and mobile phone (*r* = .15) addiction. The BWAS seems to be a promising tool, although more studies about its psychometrics in cross-cultural samples are needed.

In addition, several other scales to measure workaholism exist but have received little, if any, empirical attention compared to the aforementioned scales. Among these is an 18-item forced choice scale called *Schedule for Nonadaptive and Adaptive Personality – Workaholism* (SNAP-Work; Clark, 1993; Clark, Livesley, Schroeder & Irish, 1996). SNAP is based on trait theory and clinical psychopathology, and assesses workaholism as a personality trait/disorder. It covers work issues (13 items; e.g., “People say I neglect other important parts of my life because I work so hard”) and obsessive–compulsive traits (five items; e.g., “I don’t consider a task finished until it’s perfect”), and correlates positively with WorkBAT-Drive (McMillan et al., 2002). Also, Mudrack and Naughton (2001) developed the *Non-Required Work Scale* (four items; e.g., “Thinking of ways to improve the quality of work provided to customers and/or co-workers”) and *Control of Others Scale* (four items; e.g., “Fixing problems created by other people”). All eight items are answered on a 5-point Likert scale. These scales were based on Scott et al.’s (1997) conceptual definition of workaholism, thus conceptualizing workaholism as observable behavioral tendencies. Furthermore, as part of the *Shorter PROMIS Questionnaire* (SPQ; Christo et al., 2003), 10 items answered on a 6-point Likert continuum measures addiction to work (e.g., “Others have expressed repeated concern over the amount of time I spend working”). The theoretical underpinning is a comprehensive conceptual model of addiction. The *Children of Workaholic Parents Screening Test* (CWST) was developed by Robinson and Carroll (1999) to measure how workaholics’ adult offspring perceive their parents’ behavior (parental workaholism). CWST consists of 30 forced choice items similar to the WART. Finally, the 72 items *Workaholic Adjective Checklist* (WAC; Haymon, 1992) (e.g., “I often think my work is not as good as it could be”) and *Work Attitudes Questionnaire* (WAQ; Doty & Betz, 1981) have been developed. WAQ and WAC assess workaholism as an attitude and/or observable behavior. Overall, most of these measures have been psychometrically evaluated to little extent or not at all.

**Table 1.** Overview of particular workaholism measures

Instrument	Background/Conceptual model	Items	Subscales	Scoring/cut-off	Sample and statistical methodology^*^	Comments
Workaholism Battery (WorkBAT) (Spence & Robbins, 1992)	Based on an atheoretical approach – on attributes from the literature and the creators own hypothesis Measures 2 types of workaholism: non-enthusiastic also called “real” workaholics, and enthusiastic workaholics Measures workaholism as an attitude/obsession-compulsion	25, 24, 20, or 14	Work Involvement (WI), Drive (D), Work Enjoyment (WE)	– 5-point scale - 1 = strongly disagree, 5 = strongly agree - Score range: 25-125 - Summed total and summed subscale totals - Cut-score: Above mean on WI and D/below mean on WE for non-enthusiastic workaholics; above mean on all three subscales for enthusiastic workaholics	291 U.S. social workers Analysis of internal consistency Cluster analysis One-way analysis of variance Correlation analysis	Controversy over dimensionality WI shows poor psychometric properties D and WE good empirical support Some argue that WE is irrelevant Widely used and psychometrically tested
Work Addiction Risk Test (WART) (Robinson, 1989, 1999)	Based on an atheoretical approach – on symptoms reported by clinicians treating workaholics Measures workaholism as a Type-A behavior rather than an addiction	25, 15, or 9	Compulsive Tendencies (CT), Control (C), Impaired Communication/Self-Absorption (IC/SA), Inability to Delegate (ID), Self-Worth (SW)	– 4-point scale - 1 = never true, 4 = always true - Score range: 25-100 - Summed total and summed subscale totals - Cut-score: >57-66 = moderately work-addicted; 67-100 = highly work-addicted	363 U.S. college students Analysis of internal consistency One-way analysis of variance Correlation analysis	Originally seen as uni-dimensional but later discovered as a five dimensional model with three main factors Widely used and psychometrically validated (primarily by original author)
Dutch Work Addiction Scale (DUWAS) (Schaufeli, Shimazu & Taris, 2009)	Based on items from WART-CT and WorkBAT-Drive Measures workaholism as an excessive obsessive-compulsion	10 or 17	Working Excessively (WE), Working Compulsively (WC)	– 4-point scale - 1 = (almost) never, 4 = (almost) always - Score range: 10-50 - Summed total and summed subscale totals - Cut-score: >75th percentile	10,905 Dutch/Japanese employees Exploratory factor analysis Confirmatory factor analysis Analysis of internal consistency Odds ratio analysis Correlation analysis	Psychometrically validated Brief
Bergen Work Addiction Scale (BWAS) (Andreassen et al., 2012)	Based on Brown’s (1993) behavioral addiction components and Griffiths’ (2005) components model of addiction. Items worded in line with diagnostic addiction criteria in DSM. Measures workaholism as an addiction	7	None	– 5-point scale - 1 = never, 5 = always - Score range: 7-35 - A composite score is calculated by adding the scores on the 7 items - Cut-score: >4 on at least 4 of 7 criteria (polythetic cut-off)	12,137 Norwegian employees Item selection analysis Confirmatory factor analysis Analysis of internal consistency One-way analysis of variance Chi-square analysis Correlation analysis	Psychometrically validated Unidimensional model Solid theoretical underpinnings Brief

^*^ Sample and statistical methodology used in the initial scale-construction key studies.

In sum, the workaholism measuring tools emphasize different features of workaholism (see Table 1). Some operationalize workaholism as an attitude, a behavior (e.g., WAQ, WAC), or a trait (e.g., SNAP-Work), some as a compulsion and/or obsession (e.g., DUWAS), while yet others emphasize workaholism as an addiction (e.g., BWAS). Most of the scales assess workaholism as a multidimensional concept (e.g., DUWAS, WART, WorkBAT), while a few measure it along one dimension (e.g., BWAS). Some are particularly applicable for epidemiological studies due to their brevity (e.g., BWAS, DUWAS). Although several measures of workaholism have been developed over the years, the vast majority of these are constructed using a seemingly atheoretical approach, lacking a firm theoretical anchoring, and showing poor convergent validity with each other (Andreassen et al., in press). A reliable and valid measure of workaholism is critical for researchers and practitioners to accurately identify workaholics, to rate the magnitude of the behavior, to address the causes and consequences of workaholism, and to plan interventions.

## Antecedents

Workaholism is a complex phenomenon that is likely triggered and maintained by a diversity of factors. In the following paragraphs some central theories are presented explaining what drives people into workaholism.

Workaholism has been explained by inner and outer work motivational forces (Burke & Matthiesen, 2004; McMillan et al., 2002; van Beek, Hu, Schaufeli, Taris & Schreurs, 2012). The phenomenon can relate to internal *basic psychological needs* for autonomy, competence, and relatedness that are regarded as sources of all human behavior (Deci & Ryan, 2000). Workaholism can be connected to these needs. When one feels incompetent one can, for example, work hard for the purposes of feeling competent, especially if this motive is highly prioritized by the person in question. Internal pressure or obsession with work can thus be related to unsatisfied needs that are sought to be filled or met though work. In line with this, Andreassen, Hetland and Pallesen (2010) found that compulsive work drive was associated with unsatisfied needs. Other studies show that workaholism is also related to external reinforcement or behavior regulation (e.g., acknowledgement from others and avoidance of criticism from others) (van Beek et al., 2011, 2012). According to these studies engaged workaholics are driven to a greater extent by internal reinforcement/behavior regulation in line with own values, goals, and interests.

Workaholism is regarded by many as a *personality trait*. Particularly high scores on traits such as neuroticism, conscientiousness, narcissism, and perfectionism relate to workaholism (Andreassen, Ursin, Eriksen & Pallesen, 2012; Andreassen et al., 2010; Burke, Matthiesen & Pallesen, 2006; Clark, Lelchook & Taylor, 2010).

Cognitive perspectives have also been used to understand the phenomenon. *Basic cognitions* (schemata, assumptions, expectations, attributions, automatic thoughts) are assumed to activate behavior (Beck, 1995). Thus, if a person has a low self-image, and has a basic belief that hard work spells success, the person may then exhibit workaholic behavior. *Positive self-efficacy* (Bandura, 1986) may also be a relevant explanatory factor. If positive efficacy at work is better than in settings outside work, it can drive the person to prioritize work tasks. The cognitive perspective on workaholism has been tested empirically to a certain extent. Burke et al. (2006) found that workaholism was related to high generalized self-efficacy. In addition Andreassen, Hetland and Pallesen (2012) recently showed that passive avoidance and depressive reaction pattern (negative or no outcome expectancy) were linked with obsessive work drive. Furthermore, Graves, Ruderman, Ohlott and Weber (2012) concluded that work drive was negatively related to self-esteem.

Researchers in the field of addiction claim that workaholism has clear similarities with other *addictive behavior* – seen in light of both medical and psychological explanatory models (Eysenck, 1997; Goodman, 1990; Griffiths & Karanika-Murray, 2012; Sussman, 2012). The *medical models* emphasize physical dependence on a substance (internal or external) characterized by increased tolerance, withdrawal and craving when supply of the substance decreases, is absent or is removed (Brown, 1993). It has likewise been claimed that workaholic behavior is stimulated by physical activation produced by, for example, working hard to meet deadlines (Fassel, 1990). With reference to the *psychological addiction models,* it has also been suggested that workaholism may be developed and maintained by a hunger for appraisal, reward and reputation rooted in narcissistic traits (Andreassen, Ursin et al., 2012).

According to learning theory (Skinner, 1974), workaholism can be explained by ordinary *learning principals.* Provided the right conditions are present, this entails that anyone can become a workaholic. In *operant conditioning,* workaholic behavior occurs and recurs because similar previous behavior has led to positive outcomes (e.g., praise from colleges, promotion, salary increase) or because the behavior has resulted in avoidance of negative outcomes (e.g., criticism from leader, conflicts at home, boring leisure). In line with this, workaholism has previously been linked to salary raises and promotions (Burke, 2001). *Social learning* (Bandura, 1986) can also explain the behavior: a person is influenced by observing the behavior of significant others (e.g., parents, colleges, managers) and by exposure to role models in the media. Such views on workaholism have been explored to little extent (McMillan, O’Driscoll & Burke, 2003).

Some researchers think workaholism is best viewed from a *family* perspective (Robinson, 2013), where certain types of family dynamics (e.g., over responsibility) influence individuals within the system (Hayes, 1991; Robinson, 2013). In some cases, long work hours can be motivated by a desire to take care of one’s family, which in turn reflects over-responsibility. In one study, it was found that students with high scores on workaholism perceived their parents as more hard-working than did students with lower scores on workaholism (Chamberlin & Zhang, 2009). Whatever the case may be, like the other theories discussed so far, the empirical foundation for this perspective is equally sparse (McMillan et al., 2003).

Although the theories presented here explain workaholism differently, they are not mutually exclusive. Workaholism is probably a result of *predipositional factors* (e.g., needs, values, traits, genes), *socio-cultural experiences* (e.g., social learning, culture emphasize on competence and competition) and *behavioral reinforcements* (e.g., organizational reward systems, satisfaction, complaints and compliments) (Ng et al., 2007).

## Consequences

Workaholism can negatively influence private relations, leisure and health (Andreassen, Griffiths, Hetland et al., 2012). The symptoms are similar to what we see in other addictions, including effects on mood, tolerance and withdrawal (Andreassen, Griffiths, Hetland et al., 2012; Griffiths, 2011; Sussman, 2012). The following presents an overview of central findings related to the consequences of workaholism.

Studies show that when one experiences stress at work, individuals with a strong internal work drive report an increase in subjective *stress-related physical* and *psychological symptoms* compared to those with low scores (Andreassen et al., 2007; Andreassen, Hetland, Molde & Pallesen, 2011; Bonebright et al., 2000; Schaufeli et al., 2008; Taris, Schaufeli & Verhoeven, 2005). Thus, it is possible that *coping style* regulates the relationship between workaholism and health (Andreassen, Hetland et al., 2012; Shimazu, Schaufeli & Taris, 2010).

Two studies have looked further into how workaholism relates to sleep. In one study, persons with the highest scores on workaholism were more likely than workers with low scores to report *sleep problems, tiredness at work,* and *difficulties waking up* in addition to *fatigue* in the mornings (Kubota et al., 2010). In the other study, Andreassen et al. (2011) found that high obsessive work drive was associated with insomnia.

Furthermore, research shows that workaholics report more *work–family conflicts* and *poorer functioning outside work* than non-workaholics (Andreassen, Hetland & Pallesen, 2013; Bonebright et al., 2000; Russo & Waters, 2006; Taris et al., 2005). Since time is a limited resource, it is natural that workaholism has an impact on the domestic front. Few studies, however, have been conducted with a closer examination of how workaholism affects the work–family relationship. In a recent study that differentiates between positive and negative spillover it was found that obsessive work drive was linked to negative spillover between work and family (Andreassen, Hetland et al., 2013).

Taken together, it seems that the core element of workaholism, *internal obsessive work drive,* is associated with several negative symptoms (Andreassen et al., 2007, 2011; Andreassen, Griffiths, Hetland et al., 2012; Andreassen, Griffiths et al., 2013; Andreassen, Hetland et al., 2012; Bonebright et al., 2000; Schaufeli et al., 2008; Shimazu & Schaufeli, 2009; Shimazu et al., 2010; Taris et al., 2005). Work drive has also been linked to *lower life* and *job satisfaction* (Andreassen et al., 2011; Bonebright et al., 2000). In addition, it has been demonstrated that workaholism is associated with *reduced psychological well-being, perceived health, happiness* (Chamberlin & Zhang, 2009; del Libano et al., 2010), and *self-reported work performance* (Shimazu & Schaufeli, 2009).

## Treatment

No randomized, controlled studies on treatment of workaholism have been conducted so far, but several recommendations for treatment approaches have been proposed. These are discussed below.

A systematic review of the literature shows that *Cognitive Behavioral Therapy* (CBT) is *per se* the most well-documented and effective treatment approach for behavioral addictions (Pallesen, Mitsem, Kvale, Johnsen & Molde, 2005). The main element is cognitive restructuring and introduction of thoughts and behavior that reduce relapse. CBT typically entails helping the workaholic to set limits by, for example, using time-management principles. Within CBT, *Rational Emotive Behavioral Therapy* (REBT; Ellis, 1957) is a used method for treatment of workaholism (Chen, 2006). The essence is the ABC-theory, where A stands for the activating event, B the belief, and C the emotional or behavioral consequence. The conversations usually aim at discussing irrational beliefs/assumptions (“I must finish work myself, as no one else does it right”) and replace absolute terminology, such as the terms *must, should* or *shall* with more nuanced words. Rational emotive imagery is also an applicable approach where one asks the person to imagine a bad work-related scenario. Thereafter the person is guided to change his/her negative emotional reactions to the scenario into more constructive and positive feelings, and thus become more aware of his/her own capacity and potential for positive changes. Role-playing is often used to teach the workaholic how s/he can cope with unpleasant feelings and the irrational belief/assumption (Chen, 2006).

One recommended treatment method for other addiction behavior is *Motivational Interviewing* (MI) (Lundahl, Kunz, Brownell, Tollefson & Burke, 2010; Miller & Rollnick, 2012). The method can be used separately or together with other treatment forms. MI consists of some (1) ground principals (show empathy, develop discrepancy, role with resistance, avoid argumentation and confrontation, support efficacy), (2) communication skills (open questions, affirmations, reflections, summations), and (3) strategies (explore the problem, add knowledge, ambivalence exploration, scaling change motivation, make a menu of actual problematic issues and, together with the client, set an agenda for the conversation). In Motivational Interviewing it is thus not recommended to tell the workaholic to cut down on working hours, as this will create resistance and slow down the change-process. Instead, focus is on bringing forward the persons own thoughts, where the subject discovers the negative sides of his or her present work behavior. The content of the conversation is adjusted in accordance with how ready the person is for change, and can be used in meetings with all subjects, from the top motivated to the most unmotivated person. Triggering and emphasizing *change talk* is central in MI. Verbal statements entailing reasons, needs, desires, and ability to change behavior are examples of change talk (e.g., “I should do something before my health breaks down” or “I wish to spend more time with my children”). It is assumed that the workaholic is influenced by hearing herself/himself say and present his/her own change talk and that this helps even more to strengthen and facilitate the change-process. Thus, the focus is on how the workaholic wishes her/his life situation to become and what s/he can do to get there. MI generally aims at increasing and strengthening the person’s intrinsic motivation for change.

A third meaningful method is to approach the issue with *positive psychology* (Burnwell & Chen, 2008) – this means emphasizing strengths and positive human qualities rather than shortcomings and problems. These techniques entail, among other things, a focus on self-caring (exercise, rest), quality time (relax alone) and development of a guiding vision about what is most meaningful in life. Practice and teaching in recovery processes can also be helpful for many (Binnewies & Sonnentag, 2008).

Finally, *Anonymous Workaholics,* who base their agency on the well-known 12-step program, are established worldwide via on- and offline meetings (Fry, Matherly & Vitucci, 2006). Even when ordinary meetings are not located where one lives, people can participate in meetings via the Internet or call a helpline anonymously around the clock (http://www.workaholics-anonymous.org/pdf_files/24hourphoneline.pdf).

## Organizational Interventions

Modern work life often seeks and selects workers who are highly motivated and committed – willing to work hard and to go the extra mile. With this kind of workforce, it is an advantage to be able to differentiate between work engagement and workaholism and be conscious of interventions against workaholism on the organizational level. Employers, for example, can establish norms and values that cater to work engagement and efficiency instead of workaholism. This can be done, for example, by making sure that the workplace is a source of satisfaction of basic internal *psychological needs* (e.g., autonomy, competence, relatedness). Specifically, the employer can (1) establish a good balance between effort and reward, (2) give stimulating and optimal challenging work tasks, (3) give continuous and constructive feedback, and (4) define the employees’ jobs in terms of future prospects and security. In such interventions, management leaders play a crucial role. Thus, the employer can emphasize *leadership development,* where leaders are trained to recognize and act in line with the employees’ needs as well as their own, and reap the harvest of success together as a team.

In addition, managers should be aware of the importance of as the image they project as role models, especially since some studies show that workaholism is more prevalent among managers than others (Andreassen, Griffiths, Hetland et al., 2012; Andreassen, Ursin et al., 2012; Taris et al., 2012). Generally, managers should be aware of how they lead their followers. A great body of leadership research shows that *transformational leadership* (e.g., influences through image, uses inspirational motivation, shows individual consideration, intellectually stimulates followers) is linked to productivity and satisfaction among employees, while the contrary is the case for laissez-faire (passive-avoidant) and unfair leadership (Hetland, Hetland, Andreassen, Pallesen & Notelaers, 2011; Hetland & Sandal, 2003). By virtue of the way leaders communicate – intentionally or unintentionally – their priorities, values and presumptions to the remaining co-workers, they have either a positive or a negative influence. In turn, the employees pick up on the focus of attention in management; it can measure, control, motivate or demotivate an organization. If workaholic behavior is rewarded (e.g., complimented, salary increase, promotion) it can create a fertile environment for workaholism. If, however, excessive and obsessive working is not met with a positive reaction, workaholic behavior will be dampened. Based on this, employers should carefully consider their *reward system.*

Some organizations arrange *work–life balance programs* where they offer their employees training, for example, in time management, stress and relaxation techniques and in setting boundaries. Thereby a clear message is sent that the employee’s health and wellbeing are of vital importance and are highly prioritized by the organization. One study emphasizes that *flexible working hours* reduced reported work–family conflicts for some types of workaholics (the enthusiastic), but not for others (the non-enthusiastic) (Russo & Waters, 2006). Thus, flexible working hours do not always serve their intended purpose for everyone and can, in some cases, cultivate workaholism even more.

Finally, it should be mentioned that employers can also offer *career counseling* by using internal or external consultants. Such counseling may entail changes in the work content, establishing meaningful relations and stimulation for the employee as well as improving the relationship between the employee and the work assigned to be done.

## Future Research Directions

This field of research has yet to resolve many important issues. The prevalent use of cross-sectional study designs makes it hard to reveal potential cause-and-effect relations, for example between workaholism and health-related problems. No studies have used objective register data. In many countries there is ample opportunity for conducting such studies because data from surveys can be linked to health registers through personal ID numbers. As such, one can assess whether workaholism is connected to negative outcomes over time (e.g., heart- and cardiac system disease). In addition, very few studies have collected collateral information (e.g., partner, college). By using this, one can investigate the perceptual processes associated with workaholism. Such data can also contribute to assessing the potential impact on others, for example the family.

Another weakness is tied to the definition, operationalization, and ambiguity of workaholism as a concept. The majority of the assessment tools developed are only vaguely embedded within firm theoretical frameworks. Also, they have seldom been validated against each other or other tools that measure similar phenomena, such as obsessive job passion (Vallerand et al., 2010) and work over-involvement (Lehr et al., 2010).

A more general consensus on operationalization will facilitate a crosswise comparison of study findings. Such a consensus will also facilitate norm setting and determination of appropriate cut-scores for workaholism versus non-workaholism. This will also facilitate measurement of the effect of interventions. Moreover, the establishment of cut-scores will enable researchers to better estimate the occurrence of workaholism in the general population worldwide.

Almost without exception, all research on workaholism has so far been built on surveys. We are not aware of any studies investigating biological correlates of workaholism, for example, being genetically prone/exposed (Lobo & Kennedy, 2006). Furthermore, and in contrast to research on other addictions (Hønsi, Mentzoni, Molde & Pallesen, in press), no studies have explored whether workaholism influences cognitive processes such as attentional bias. Controlled intervention studies on workaholism are totally absent in the field. Thus, development of interventions and studies of their effects should also be prioritized in the future.

Generally speaking, future research efforts in this field should focus on longitudinal designs, incorporating objective and collateral parameters of behavior and health anchored in firm theoretical models.

## Conclusions

This article provides an overview of the phenomenon of workaholism (stable tendency to compulsively and excessively work) (see Figure 1). Although the workaholism construct has become a part of our everyday vocabulary, we still know little about the scientific basis for it. Reasons why some people become workaholics are probably numerous and complex. Generally, studies show that workaholism is equated with negative outcomes. To date, no documented treatment for workaholism exists. The field is in great need of longitudinal studies including objective parameters of behavior and health.
